# Epidemiological Investigation of Peste des Petits Ruminants in Bahrain

**DOI:** 10.3390/v18060634

**Published:** 2026-05-31

**Authors:** Ahmad Almajali, Shereen Adel Al Kazaz, Zainab Abdulhussain Mohammed, Mohammed Hamdy Mohammed, Hassan Jawad Al Hashim, Ali Hussain Makki, Fajur Sabah Al Saloom, Abbas Al Hayki, Markos Tibbo

**Affiliations:** 1 Subregional Office for the Gulf Cooperation Council States and Yemen, Food and Agriculture Organization of the United Nations (FAO), Al Qala-id Street, Abu Dhabi P.O. Box 62072, United Arab Emirates; 2 Department of Veterinary Clinical Sciences, Faculty of Veterinary Medicine, Jordan University of Science and Technology, Irbid 22110, Jordan; 3 Regional Representation for the Middle East, World Organization of Animal Health, Beirut P.O. Box 901965, Lebanon; 4 Veterinary Laboratory, Ministry of Municipalities Affairs and Agriculture, Manama P.O. Box 251, Bahrain; salkazaz@mun.gov.bh (S.A.A.K.); zmohamed@mun.gov.bh (Z.A.M.); fsalman@mun.gov.bh (F.S.A.S.); 5 Veterinary Service, Budaiya, Ministry of Municipalities Affairs and Agriculture, Manama P.O. Box 251, Bahrain; mhamdi@mun.gov.bh (M.H.M.); halhashim@mun.gov.bh (H.J.A.H.); ahmhasan@mun.gov.bh (A.H.M.); aaalhayki@mun.gov.bh (A.A.H.)

**Keywords:** small ruminants, Peste des petits ruminants, epidemiology, Bahrain

## Abstract

Peste des petits ruminants (PPR) is a highly contagious transboundary disease that affects small ruminants and impacts livestock production and trade. This study investigated the seroprevalence and associated risk factors of PPR in sheep, goats, camels, and wild ruminants in Bahrain. A total of 1240 sheep, 1224 goats, 100 camels, and 38 wild ruminants were tested using competitive ELISA. The individual seroprevalence rates were 26% in sheep and 25.5% in goats, with flock/herd-level prevalences of 22.7% and 29.6%, respectively. No antibodies were detected in camels or wild ruminants. The highest seroprevalence was observed in the Northern governorate. The identified risk factors included geographic location, age (<12 months for goats), sex (male for goats), and health status (weak animals). At the flock/herd level, large flock size and semi-intensive farming increased the likelihood of seropositivity. In addition, a 2023–2024 surveillance campaign tested 1044 young, locally born lambs and kids across all governorates. All animals were found to be negative for PPR according to a competitive enzyme-linked immunosorbent assay (cELISA) and a reverse transcription polymerase chain reaction (RT-PCR) test, confirming the absence of antibodies and active virus circulation in the population. These findings support the classification of Bahrain at Progressive Control Pathway for PPR (PCP-PPR) Level 3 status and emphasize the importance of continued surveillance and regional cooperation to mitigate the spread of diseases.

## 1. Introduction

Peste des petits ruminants (PPR) is a major transboundary animal disease that causes significant direct and indirect economic losses owing to high mortality and impairment of livestock trading [1,2,3]. PPR is an acute viral disease that primarily affects domesticated and wild small ruminants. Clinically, PPR is characterized by fever; pneumonia; necrosis; ulceration of mucous membranes, especially in the buccal cavity; and inflammation of the gastrointestinal tract, leading to severe diarrhea [4].

The PPR virus is an RNA virus that belongs to the *Morbillivirus* genus and is antigenically related to the Rinderpest virus, which infects large ruminants [5,6]. Based on the partial sequence of the fusion (F) gene, Peste des petits ruminants virus (PPRV) isolates were grouped into four lineages. Lineage III form of PPRV was mainly reported in Africa, whereas most isolates from Asia, mainly the Middle East, were of Lineage IV [7,8,9,10,11,12].

The first case of a PPR outbreak was reported in West Africa in the early 1940s and later recognized as an endemic disease in all countries in West and Central Africa [13]. PPR is endemic in most Middle Eastern countries and is mainly controlled by yearly vaccination with a modified live vaccine containing the Nig75/1 isolate [14,15,16].

The first and only outbreak of PPR reported in two governorates (the Northern and Al-Muharraq governorates) of Bahrain occurred in imported sheep flocks in 2012 [17]. Vaccination has never been practiced and is not allowed by law in Bahrain. Similar to other Gulf Cooperation Council (GCC) states, Bahrain follows the Progressive Control Pathway for PPR (PCP-PPR) agreed under the FAO-WOAH Global Framework for the Progressive Control of Transboundary Animal Diseases (GF-TADs) and is currently ranked at Level 3.

The objectives of this study were to investigate the prevalence of PPR in sheep, goats, camels, and wild ruminants in Bahrain and to determine the risk factors associated with seropositivity to PPR. To complement previous serological studies conducted in 2021, a 2023–2024 surveillance campaign targeting young, locally born lambs and kids was conducted. This effort aimed to detect active circulation of the PPR virus in the absence of vaccination.

## 2. Materials and Methods

### 2.1. Study Design and Population

A cross-sectional study was conducted to investigate the seroprevalence and active circulation of PPR among livestock in Bahrain. This study included two components: (1) a serological survey conducted in 2021 (from September to December 2021) targeting sheep, goats, camels, and wild ruminants and (2) a surveillance campaign conducted from September 2023 to August 2024 targeting young, locally born lambs and kids. This surveillance campaign aimed to detect active PPR virus circulation in the absence of vaccination. Bahrain has four governorates: the Capital, Muharraq, Northern, and Southern governorates. Bahrain’s livestock production systems include traditional and semi-intensive farming, with animals primarily maintained indoors under zero-grazing conditions. Vaccination against PPR is prohibited by law, which provides a unique environment for assessing natural disease dynamics. A limited number of wild ruminants drawn from a very small, confined, and managed population of wild ruminants (estimated at 600 heads) in the protected Al Areen Wildlife Park in Bahrain, were also sampled. As such, our sample proportionally represents the accessible population.

### 2.2. Sampling

Each governorate was considered an independent epidemiological unit. For sheep and goats, the sample size was determined using a 95% confidence interval, a 5% margin of error, and an assumed prevalence of 50%. In contrast, the wild ruminant sample represented the accessible managed population in Bahrain and was not intended as a statistical extrapolation. The Kingdom of Bahrain is endowed with an estimated 85,000 small ruminants, comprising approximately 60,000 sheep and 25,000 goats, alongside a camel population of about 3000 [18]. A total of 1240 sheep, 1224 goats, 100 camels, and 38 wild ruminants were randomly selected. For sheep and goats, stratification was performed based on flock/herd size (<50, 50–200, and >200 for sheep; <50, 50–100, and >100 for goats). Camels and wild ruminants were sampled from a single epidemiological unit because of their relatively low population numbers. A serological survey was conducted from September to December 2021.

For molecular surveillance (conducted from September 2023 to August 2024), 1044 young lambs and kids born locally and not imported were sampled from farms across all four governorate regions. The animals were selected to represent the most susceptible age group and to ensure geographic coverage. Nasal and ocular swabs were collected using sterile polyester-tipped swabs (Copan Diagnostics, Brescia, Italy), transported in viral transport medium at 4 °C, and processed within 24 h.

The herds/flocks to be sampled were randomly selected using the computation function of the SPSS software (SPSS v. 25, IBM, Chicago, IL, USA). Blood was collected from the jugular vein of the selected animal and transported in a cold box to the laboratory, where the sera were isolated and stored at −20 °C until further analysis. A pre-tested semi-structured questionnaire was administered to collect health and management information about each sampled herd/flock. Health information included the presence of clinical signs of PPR, mortality rate, abortion rate, and vaccination history. Management information included the source of water, disinfection and cleaning practices, and workers’ farming behaviors.

### 2.3. Laboratory Analysis

Serum samples from the serological survey were screened for PPR antibodies using a competitive enzyme-linked immunosorbent assay (cELISA) (Innovative Diagnostics, Lyon, France). The test included strong positive, weak positive, and negative controls, as provided by the manufacturer. The sensitivity and specificity of the cELISA were 94.5% and 99.4%, respectively, compared to virus neutralization assays [19].

Serum and swab samples collected during the 2023–2024 surveillance campaigns were tested for antibodies and PPR viral RNA using cELISA and reverse transcription quantitative polymerase chain reaction (RT-PCR) with a commercial kit (ID Gene™, Innovative Diagnostics, Lyon, France). RNA was extracted using the ID Gene™ Spin Universal Extraction Kit (Innovative Diagnostics, Lyon, France) in accordance with the manufacturer’s instructions.

### 2.4. Data Collection and Risk Factor Analysis

A semi-structured questionnaire was administered to livestock owners to collect information on herd management, health status, mortality rate, and potential risk factors for PPR exposure. Recorded variables included age, sex, location, herd size, management practices, and disease history.

### 2.5. Statistical Analysis

All data, information, and results of the sera and information collected using the semi-structured questionnaire were coded and added to an SPSS spreadsheet. Frequency tables were produced using cross-tabulation. Univariate analysis was performed using χ^2^. Multivariate analysis was performed using regression analysis. All analyses were performed using SPSS version 25 software (IBM Corp., Armonk, NY, USA).

## 3. Results

### 3.1. Prevalence of PPR

**Figure 1:**
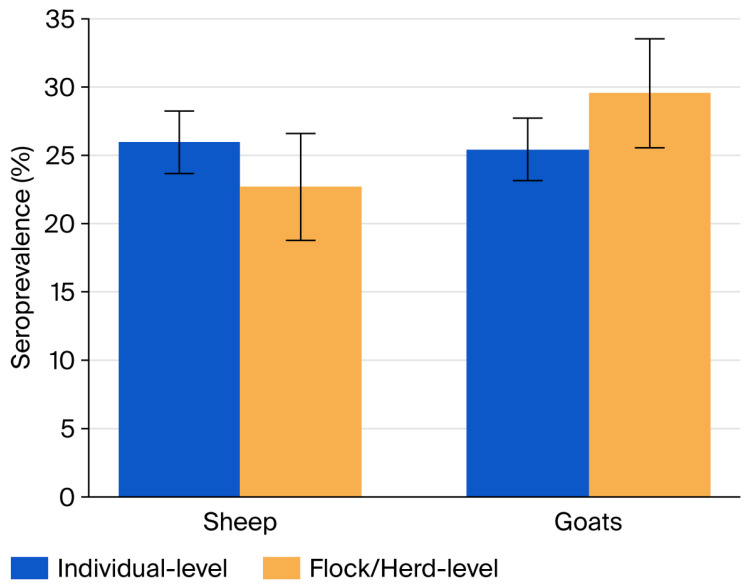
PPR seroprevalence in sheep and goats in Bahrain, as assessed in 2021.

Of the 1240 tested sheep sera, 323 (26%) were seropositive for PPR antibodies, whereas in goats, 312 of 1224 (25.5%) were seropositive (Table 1). The flock-level seroprevalence in sheep was 22.7%, whereas the herd-level prevalence in goats was 29.6%. No seropositive cases were detected among the 100 camels or 38 wild ruminants tested. Figure 1 illustrates the distribution of seropositive cases across the different governorates.

**Figure 2:**
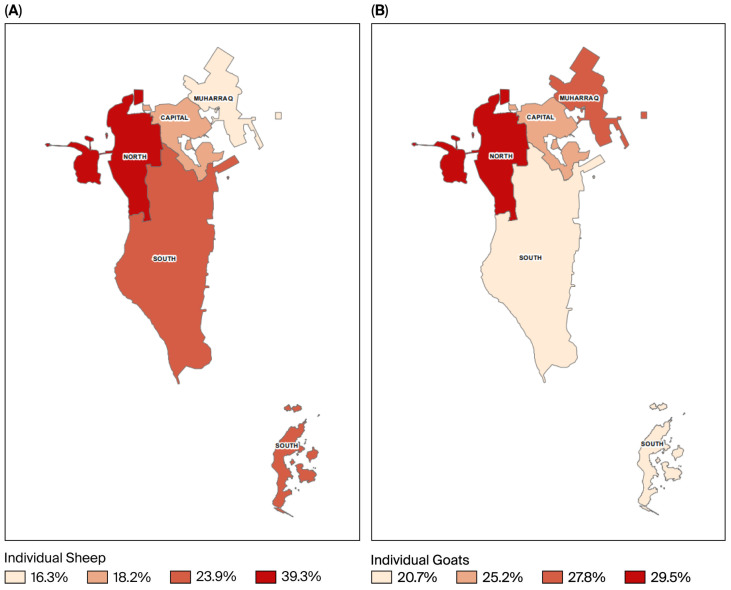
Distribution of PPR seropositive sheep (**A**) and goats (**B**) in the different governorates of Bahrain.

The distribution of prevalence in different governorates is shown in Figure 2. The PPR prevalence was highest (*p* < 0.05) in sheep and goats from the Northern governorate. None of the screened camels or wild ruminants tested positive according to the cELISA, suggesting the absence of PPR infection in these animal species.

### 3.2. Risk Factor Analysis

The initial univariate analysis using the chi-square test revealed three factors that influenced the seropositivity of sheep to PPRV. Sheep located in the Northern governorate, of female sex, and of weak health are more likely to be seropositive for PPRV (Table 1).

In goats, the univariate analysis suggested that location (Northern governorate), age less than 12 months, sex (males), and weak health were risk factors associated with seropositivity to PPR (Table 2).

At the flock/herd level, the univariate analysis revealed two risk factors for sheep flocks’ seropositivity to PPR (being located in the “Northern governorates” and a large herd size) (Table 3). For goat herds, farming type (semi-intensive farming) was identified as a risk factor for PPR seropositivity (Table 4).

The multivariable logistic analysis for sheep seropositivity to PPR suggested that sheep in the Northern governorate were more likely (OR 3.07) to be seropositive than those in the other governorates. However, the multivariable analysis for goats revealed that goats from the Northern governorate (OR 2.02), those aged less than 12 months (OR 2.06), and those of male sex (OR 2.20) were more likely to be seropositive for PPR (Table 5).

A targeted serological and virological surveillance campaign conducted between September 2023 and August 2024, involving 1044 young, locally born lambs and kids across all four governorates, confirmed that all animals tested negative for PPR according to the cELISA and RT-PCR, indicating no antibodies or active virus circulation within the susceptible population. 

Table 6 summarizes the serological and molecular surveillance from 2021 to 2024 showed PPR antibodies in sheep and goats in 2021, but no seropositivity or viral RNA in young animals tested during 2023–2024.

## 4. Discussion

The epidemiology of PPR is not well understood in the Middle East, since the available vaccination procedures lack the capacity to differentiate between infected and vaccinated animals during serological screening. This study is the first to address the prevalence of PPR and risk factors associated with seropositivity to PPRV in small ruminants, camels, and wild ruminants in Bahrain. It provides valuable insights into the epidemiology of PPR in Bahrain, a country that has maintained a non-vaccination policy and is currently classified at Level 3 of the PCP-PPR. The findings have significant implications for disease surveillance, control strategies, and regional cooperation in the GCC region.

The detection of PPRV antibodies in a substantial proportion of sheep (26%) and goats (25.5%) suggests historical exposure to the virus, likely through the importation of vaccinated or previously infected animals. This is particularly evident in the Northern governorate, which showed the highest seroprevalence. The Northern governorate is the only region in Bahrain with a land border with Saudi Arabia, a country where PPR vaccination is routinely practiced. Vaccinated animals or vaccine vials may have been illegally introduced or smuggled into Bahrain, thus contributing to the observed seropositivity. Similar cross-border dynamics have been reported in other regions where informal livestock trade and movement have influenced disease epidemiology [2,19].

The King Fahd Causeway serves as Bahrain’s only land border, where animals are officially subject to quarantine, health certification, and inspection. Despite these measures, risks of illegal entry remain, particularly through informal exchanges between herders, traders, and communities. Animal population registration is managed through the Ministry of Municipalities Affairs and Agriculture’s Veterinary Information System, which records herd sizes and animal movements, yet the findings highlight significant enforcement gaps in regulating livestock movement across borders. Weak surveillance at entry points and limited capacity for traceability make it difficult to distinguish between locally raised and illegally introduced animals, thereby increasing the likelihood of transboundary animal disease incursions. Addressing these challenges requires strengthening veterinary inspection systems, enhancing coordination between customs and animal health authorities, and adopting modern technologies such as electronic identification and real-time reporting tools. Furthermore, cross-border collaboration with Saudi Arabia and other Gulf states should be intensified to harmonize vaccination policies, improve intelligence-sharing, and close loopholes that facilitate informal trade. Without such targeted interventions, Bahrain will remain vulnerable to repeated introductions of PPR and other transboundary diseases, threatening both livestock health security and broader food system resilience.

The absence of seropositive animals and active viral circulation, as evidenced by the negative cELISA and RT-PCR results (Table 6) in 1044 young, locally born lambs and kids sampled during the 2023–2024 surveillance campaign, is a strong indicator that Bahrain currently maintains a PPR-free status in terms of active infection. This supports the country’s PCP-PPR Level 3 classification and highlights the effectiveness of its surveillance and biosecurity measures. However, the presence of seropositive animals underscores the need for continued vigilance, especially given the porous nature of regional borders and the potential for reintroduction from neighboring endemic countries [17,20].

A comparative analysis with other countries in the Middle East revealed that Bahrain’s seroprevalence is moderate. Similar levels of PPR prevalence have been reported elsewhere [7,14,20,21,22,23,24]. However, Saudi Arabia has reported seroprevalence rates ranging from 37.7% to 86.1%, despite ongoing vaccination campaigns [15,25,26,27]. In contrast, Yemen has reported lower rates, similar to Bahrain, with 15% in sheep and 18% in goats [2]. These comparisons suggest that Bahrain’s non-vaccination policy, combined with strict import controls and surveillance, may be effective in limiting viral circulation. However, the risk of reintroduction remains, necessitating sustained monitoring and regional coordination. Bahrain’s non-vaccination policy contrasts with policies in Saudi Arabia and other GCC states, where routine vaccination complicates serological interpretation. Despite their differences, Bahrain has maintained moderate seroprevalence without active circulation, highlighting the effectiveness of strict biosecurity and surveillance.

This study also highlights the importance of risk-based surveillance strategies. Targeting young, unvaccinated animals for cELISA and RT-PCR testing provides a reliable method for detecting recent virus circulation. This approach, coupled with serological surveys in older animals, offers a comprehensive picture of both historical exposure and current infection status. Future surveillance efforts should consider incorporating molecular epidemiology tools, such as sequencing of detected strains, to trace virus origins and transmission pathways [11].

A risk factor analysis revealed several important associations. The higher seroprevalence in the Northern governorate aligns with its proximity to Saudi Arabia and the potential for informal vaccine introduction. Goats younger than 12 months of age were more likely to be seropositive, consistent with findings from other studies in the region [14,21,25,28]. In addition, male sheep and goats were more likely to be seropositive for PPR, which could be attributed to the importation of male sheep and goats from Saudi Arabia, Syria, Lebanon, Oman, and Jordan, where PPR vaccination is practiced. Weak health status in sheep and goats was another significant risk factor, suggesting that animals with compromised immunity may be more susceptible to infection or may have been exposed during periods of stress or poor management, which is consistent with previous reports [14,29,30,31].

At the flock/herd level, our findings agree with those of previous studies in which semi-intensive farming (for goat herds) and flock size (for sheep flocks) were identified as risk factors for seropositivity in sheep flocks and goat herds [14,20,21,28,31,32,33]. These systems may also facilitate the introduction and spread of pathogens through shared equipment, personnel, and inadequate biosecurity measures.

The absence of antibodies in camels and wild ruminants is noteworthy. Although PPRV has been detected in camels and wildlife in other regions [9,22], the current findings suggest that these species have not been exposed to the virus in Bahrain. This may reflect limited interactions between domestic small ruminants and other species, or it may indicate that the virus has not yet spilled over into these populations. Continued surveillance in these species is recommended, particularly in areas where interspecies contact is likely.

Policy recommendations emerging from this study include the need to strengthen border control to prevent the illegal importation of vaccinated or infected animals, enhance farmers’ awareness about PPR, and maintain high levels of passive and active surveillance. Regional cooperation is also essential, particularly in harmonizing surveillance protocols and sharing data through platforms such as the WOAH-WAHIS system. Additionally, exploring the feasibility of implementing DIVA (Differentiating Infected from Vaccinated Animals) strategies could enhance the precision of serological monitoring in the future [19].

Although Bahrain currently shows no evidence of active PPRV circulation, the presence of seropositive animals and regional disease dynamics necessitate sustained surveillance, policy enforcement, and regional collaboration to achieve and maintain PPR eradication. Future research should explore the genetic characterization of circulating PPRV strains to better understand transmission dynamics and inform policy decisions. Collaborative regional efforts with neighboring countries are essential for effective disease eradication. Comparisons with studies in neighboring countries suggest that Bahrain remains at a moderate risk of PPR outbreaks, necessitating sustained monitoring and preventive measures.

Another limitation of this study is the possibility of false-negative results in RT-PCR testing due to sample timing. Although the surveillance campaign specifically targeted young, unvaccinated lambs and kids to maximize the likelihood of detecting recent infections, transient viral shedding or sampling outside the peak viraemia window could theoretically result in undetected cases. Previous studies have shown that PPR viral RNA is shed for a limited period during acute infection, and the timing of sample collection strongly influences diagnostic sensitivity [4,8]. In addition, the absence of clinical signs at the time of sampling further reduces the probability of detecting active infections. Therefore, while our findings strongly suggest the absence of active PPR circulation, repeated surveillance campaigns and longitudinal sampling would provide additional confidence in excluding low-level or sporadic virus transmission.

## 5. Conclusions

This study provides comprehensive insights into the epidemiology of PPR in Bahrain. The serological findings suggest past exposure to PPRV or vaccination among sheep and goats, particularly in the Northern governorate. However, the absence of antibodies in camels and wild ruminants, along with the 2023–2024 surveillance campaign results, which found all 1044 young, locally born lambs and kids negative for PPR according to cELISA and RT-PCR, indicated no current active circulation of the virus. These results reinforce Bahrain’s classification at PCP-PPR Level 3 status and highlight the effectiveness of the existing biosecurity and surveillance measures. Continued investment in surveillance, risk-based monitoring, and regional cooperation will be essential to sustain Bahrain’s progress and to contribute to the global goal of PPR eradication by 2030.

## Figures and Tables

**Table 1:** Univariate analysis of seropositivity to PPR in individual sheep in Bahrain.

Variable		No. Negative (%)	No. Positive (%)	Odds Ratio
Governorate	Capital	279 (81.8)	62 (18.2)	3.3
	Muharraq	113 (83.7)	22 (16.3)	
	Northern	230 (60.7)	149 (39.3) *	
	Southern	295 (76.6)	90 (23.9)	
Age	<12 months	210 (76.9)	63 (23.1)	-
	12–36 months	234 (74.5)	80 (25.5)	
	>36 months	473 (72.4)	180 (27.6)	
Sex	Male	140 (69.7)	61 (30.3) *	1.4
	Female	765 (76.3)	238 (23.7)	
Health status	Excellent	288 (83.7)	56 (16.3)	1.9
	good	257 (77.2)	76 (22.8)	
	Weak	117 (64.3)	65 (35.7) *	
	Poor	40 (81.6)	9 (18.4)	

* Statistically significant based on the chi-square test (*p* < 0.05).

**Table 2:** Univariate analysis of seropositivity to PPR in individual goats in Bahrain.

Variable		No. Negative (%)	No. Positive (%)	Odds Ratio
Governorate	Capital	243 (74.8)	82 (25.2)	1.2
	Muharraq	114 (72.2)	44 (27.8)	
	Northern	263 (70.5)	110 (29.5) *	
	Southern	292 (79.3)	76 (20.7)	
Age	<12 months	172 (66.9)	85 (33.1) *	1.3
	12–36 months	228 (73.1)	84 (26.9)	
	>36 months	512 (78.2)	143 (21.8)	
Sex	Male	148 (63)	87 (37) *	2.0
	Female	742 (77.5)	215 (22.5)	
Health status	Excellent	229 (75.3)	75 (24.7)	1.2
	good	267 (73.6)	96 (26.4)	
	Weak	106 (66.3)	54 (33.8) *	
	Poor	32 (71.1)	13 (28.9)	

* Statistically significant based on the chi-square test (*p* < 0.05).

**Table 3:** Univariate analysis of seropositivity to PPR at the sheep flock level in Bahrain.

Variable		No. Negative (%)	No. Positive (%)	Odds Ratio
Governorate	Capital	35 (83.3)	7 (16.7)	3.5
	Muharraq	25 (83.3)	5 (16.7)	
	Northern	4 (44.4)	5 (55.6) *	
	Southern	28 (73.7)	10 (26.3)	
Herd size	<50	74 (84.1)	14 (15.9)	1.6
	50–200	13 (61.9)	8 (38.1)	
	>200	5 (50)	5 (50) *	
Farming	Traditional	90 (77.6)	26 (22.4)	-
	Semi-intensive	2 (66.7)	1 (33.3)	
Purpose	Trading and meat	0 (0.0)	1 (100)	-
	Breeding	92 (78)	26 (22)	
Source of water	Ground water	22 (73.3)	8 (26.7)	-
	Municipality water	70 (79.5)	18 (20.5)	
Presence of dogs	Yes	7 (70)	3 (30)	-
	No	85 (78)	24 (22)	
Use of disinfectants	Yes	4 (100)	0 (0.0)	-
	No	88 (76.5)	27 (23.5)	
Disposal of dead animals	Call municipality	77 (81.1)	18 (18.9)	-
	Incineration	2 (50)	2 (50)	
	Burial	13 (65)	7 (35)	
Presence of abortion	Yes	33 (78.6)	9 (21.4)	-
	No	59 (76.6)	18 (23.4)	

* Statistically significant based on the chi-square test (*p* < 0.05).

**Table 4:** Univariate analysis of seropositivity to PPR at the goat herd level in Bahrain.

Variable		No. Negative (%)	No. Positive (%)	Odds Ratio
Governorate	Capital	30 (71.4)	12 (28.6)	-
	Muharraq	23 (76.7)	7 (23.3)	
	Northern	31 (72.1)	12 (27.9)	
	Southern	4 (40)	6 (60)	
Herd size	<50	72 (72)	28 (28)	-
	50–100	10 (71.4)	4 (28.6)	
	>100	6 (54.5)	5 (45.5)	
Farming	Traditional	84 (73)	31 (27)	4.0
	Semi-intensive	4 (40)	6 (60) *	
Purpose	Trading and meat	0 (0.0)	4 (100)	-
	Breeding	88 (72.7)	33 (27.3)	
Source of water	Ground water	23 (65.7)	12 (34.3)	-
	Municipality water	65 (73)	24 (27)	
Presence of dogs	Yes	9 (75)	3 (25)	-
	No	79 (69.9)	34 (30.1)	
Use of disinfectants	Yes	5 (71.4)	2 (28.6)	-
	No	79 (73.1)	29 (26.9)	
Disposal of dead animals	Call municipality	68 (67.3)	33 (32.7)	-
	Incineration	5 (83.1)	1 (16.9)	
	Burial	15 (83.3)	3 (16.7)	
Presence of abortion	Yes	28 (70)	12 (30)	-
	No	60 (70.6)	25 (29.4)	

* Statistically significant based on the chi-square test (*p* < 0.05).

**Table 5:** Logistic regression/multivariable analysis * of individual sheep and goats’ seropositivity to PPR.

Animal Species	Variable	B	S.E.	Sig.	Odds Ratio
Sheep	Constant	19.010	0.402	0.000	
	Southern	−0.055	0.429	0.898	
	Northern	1.121	0.349	0.001	3.07
Goats	Constant	−1.939	0.482	0.000	
	Southern	0.590	0.320	3.398	
	Northern	0.705	0.341	4.264	2.02
	<12 months	0.724	0.237	9.371	2.06
	>36 months	0.476	0.235	4.092	
	Male	0.789	0.191	17.059	2.20

* Variable(s) entered in step 1: governorate, age, sex, and health condition.

**Table 6:** Summary of serological (2021) and molecular (2023–2024) surveillance results for PPR in Bahrain.

Year	Species	Method	Sample Size	Result
2021	Sheep	cELISA	1240	26% individuals and 22.7% of the flock seropositive
2021	Goats	cELISA	1224	25.5% individuals and 29.6% of the herd seropositive
2021	Camels	cELISA	100	0% seropositive
2021	Wild ruminants	cELISA	38	0% seropositive
2023–2024	Sheep and goats (young)	cELISA	1044	0% seropositive
2023–2024	Sheep and goats (young)	RT-PCR	1044	0% positive (no viral RNA detected)

## Data Availability

The datasets used and analyzed during the current study are available from the corresponding author on reasonable request.
